# Green revolution in electronic displays expected to ease energy and health crises

**DOI:** 10.1038/s41377-020-00455-9

**Published:** 2021-02-07

**Authors:** Yuyang Wang, Hui Nie, Jinsong Han, Yaxun An, Yu-Mo Zhang, Sean Xiao-An Zhang

**Affiliations:** 1 State Key Laboratory of Supramolecular Structure and Materials, College of Chemistry, Jilin University, Changchun, 130012 China; 2 Department of Chemistry and Biochemistry, University of California, Santa Barbara, Santa Barbara, California 93106 USA; 3 State Grid Heilongjiang Electric Power Co., Ltd, Heihe Power Supply Company, Heihe, 164300 China; 4 Jiaxing IrS Display Technology Co., Ltd, Jiashan, 314113 China

**Keywords:** Optical materials and structures, Green photonics, Lasers, LEDs and light sources

## Abstract

The technological revolution of long-awaited energy-saving and vision-friendly displays represented by bistable display technology is coming. Here we discuss methods, challenges, and opportunities for implementing bistable displays in terms of molecular design, device structure, further expansion, and required criteria, hopefully benefiting the light-related community.

With the rapid advancement of the internet, electronic information and globalization, electronic displays have revolutionized our lives^1–3^. Although they bring us amazing visuals and real-time information acquisition, the issues caused by their long-term use and subsequent waste, such as very high energy consumption, high related pollution, low energy utilization efficiency, and eye damage, are also becoming increasingly prominent. Therefore, the development of low energy consumption and user-friendly electronic displays has become a long-term goal for future global sustainable development.

Various electronic materials with bistable characteristics that have emerged in recent years have aroused people’s hope and excitement for energy-saving and user-friendly displays. Significant progress has been made in relevant organic or inorganic electrochromic (EC) materials, and some bistable electronic materials/technologies have begun to enter the display market. New design principles based on the proton-coupled electron transfer (PCET) mechanism and related bistable electronic materials are bringing energy-saving displays into an era where the comprehensive performance is closer to the actual application needs. Here we will focus on the key considerations of the design principles and strategies for further development of green technology, especially focusing on how to solve the challenges and bottlenecks faced by current EC materials and devices. Moreover, we will propose some suggestions based on cross-fusion strategies to help overcome the current status of continuous power consumption in mainstream light-emitting display technology so that this technology also exhibits a highly energy-efficient bistable performance. By doing so, we hope to inspire an increasing number of researchers to work together, to challenge many “impossible”, scientifically forbidden areas through unconventional design methods that eliminate inertial thinking.

## Bistable displays: promising power-saving display techniques

**Fig. 1:**
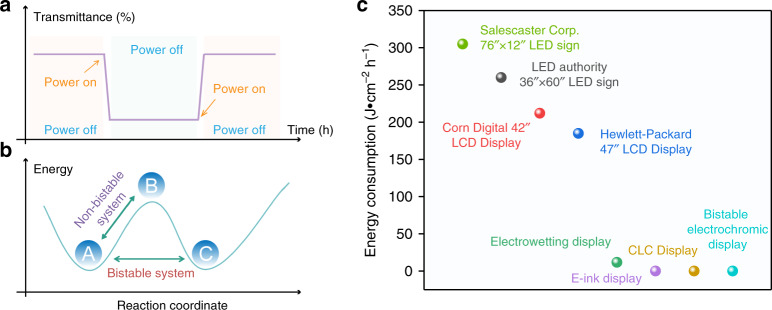
Characters of the bistable display technologies. **a** Optical characteristics of a bistable display. **b** Characteristics of bistable materials at the molecular energy level. (A stands for original state of the molecule, B and C stand for different final states of the molecule). **c** Comparison of energy consumption for different electronic displays. The energy consumption of commercialized LCDs and LEDs contains both display and heat dissipation energy consumption

How can the need for rapid economic and social development be met without aggravating harm to the natural environment and health? The development of low energy consumption and more user-friendly electronic displays is the key. Light absorption and reflection are utilized by nature for color display and environmental integration. The history of biological evolution proves that this is the best approach to utilize and transform external energy (light) (with the lowest energy consumption and the most efficient energy conversion). Therefore, “materials and technology for reflective displays” open another door for the development of electronic displays for humans. Such technology does not emit light itself but uses sunlight or external light sources to illuminate the display. This might require very little electric drive to switch pages without consuming additional power to continuously display information or images—chameleon-like “bistable” characteristics (Fig. 1a, b). This means that such strategies can greatly reduce the power and heat consumption of digital displays compared to mainstream displays (Fig. 1c), which provides a solid foundation for future low-energy displays. More importantly, the “reflective display” has a natural color mode (light scattering type) that human beings are inherently familiar with and gives users more adaptive color softness and eye comfort. The pioneering reflective display techniques include the use of cholesteric liquid crystals (CLCs)^4^, electronic ink (E-Ink)/e-paper^5,6^, electrowetting (EW)^7,8^, and electrochromism^9–13^. Taking E-Ink as an example, it is based on a microencapsulated electrophoretic technique. Under the action of E-fields, white or black microcapsules with different charges migrate up and down to display white/black images^14^. Currently, various E-Ink products, such as Kindle E-readers, billboards, and supermarket price tags, are gradually entering the world. Obviously, at this stage, E-Ink, which usually appears black and white with a slow refresh rate, cannot provide all the functions that Liquid Crystal Display-Light Emitting Diode/Organic Light Emitting Diode (LCD-LED/OLED) can. However, in some areas, low power consumption is preferable to dazzling color fast refresh, such as in the wearable industry. Recently, E-Ink and Wacom disclosed a new type of color e-paper. However, because the addition of pigments slows down the refresh rate, it might be a long time until we see color on an E-reader. CLC molecules exhibit a bistability property due to their two stable states, including the planar state and focal conic state, which can be maintained without energy consumption. The two states can be switched between under an external voltage, resulting in a system change between two states, including light and dark states. Nevertheless, CLCs usually act as light shutters. Without the help of color filters or continuously changing background light, it is very difficult to achieve multicolors, which is inconsistent with an ideal energy-saving display^15^. In EW, a highly promising display technology, an electric field drives changes in the wettability and contact angle of ink droplets on insulating substrates to display information, thereby regulating the diffusion and contraction of ink droplets by a voltage. The color pixel response time is very fast (<10 ms), which is sufficient to show video content on information displays and is shorter than that of EC/E-ink and most LCDs. However, this technology is not yet mature and it does not have bistable or completely colorless characteristics, because the color of the ink droplets remains, which limits the industrialization of EW display technology^16^. Benefiting from the relative numerous EC materials, facile design and preparation process, and coloration mechanism, EC displays have the advantages of broad color variation and high color purity compared with reflective display technologies, with great potential in future information display applications. Moreover, some intriguing EC device (ECD) structures and applications, such as EC batteries, have been developed to further promote the development of energy storage and energy-saving technologies^17,18^.

EC displays based on inorganic and organic EC materials are one of the most promising technologies for bistable displays^19–22^. They avoid the insurmountable viewing angle range limitation and the polarizers and material polarization required for LCDs. Current EC materials mainly include organic materials represented by viologens and conjugated polymers, which exhibit color changes along with variations in the electron cloud and structure caused by electron transfer; inorganic materials represented by WO_3_, NiO, and so on, which exhibit color variations together with crystal structure changes due to electron transfer and ion migration; and organic–metallic hybrid materials represented by Pt, Os, and Ir complexes, which exhibit color changes caused by electron transfer from metal ions to ligands. Among known promising EC materials, inorganic materials, mainly referring to inorganic transition metal oxides represented by WO_3_^23,24^, have been widely studied. When a voltage stimulation is applied, WO_3_ undergoes reduction/oxidation, accompanied by embedding/de-embedding of cations in its crystal lattice, thereby changing the crystal structure and color of WO_3_. Under electrical stimulation, a reversible color change of WO_3_ from colorless to blue can be achieved. The reaction can be described as follows:

$${\mathrm{WO}}_3 + {\mathrm{xM}}^ + + {\mathrm{xe}}^ - \to {\mathrm{M}}_{{x}}{\mathrm{WO}}_3$$

**Fig. 2:**
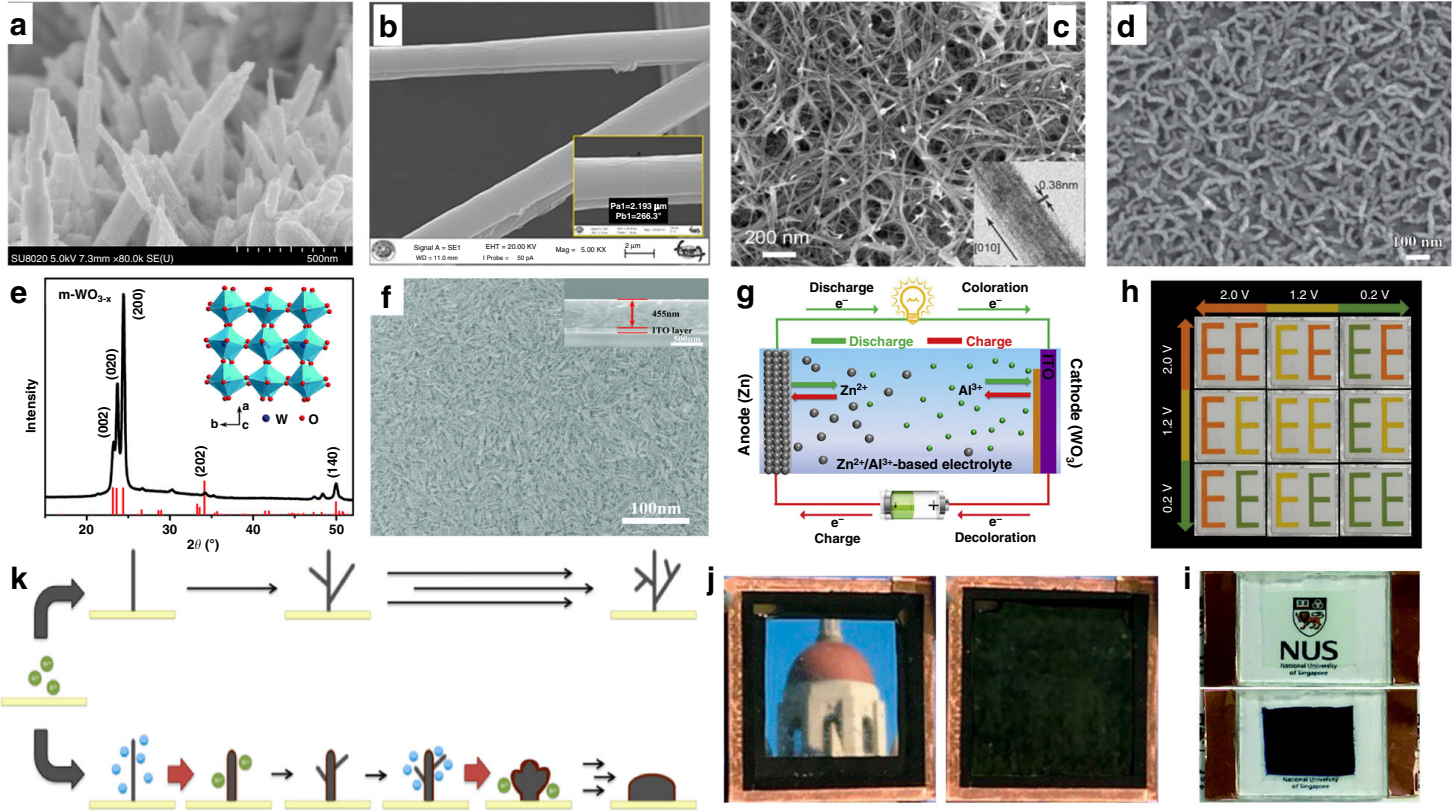
Latest developments in various inorganic electrochromic materials and devices. **a** Scanning electron microscopy (SEM) image of WO_3_ nanorods^79^. **b** SEM image of PPy/WO_3_/BMIMBF_4_ nanofibers^80^. **c** Typical SEM image of a WO_3_ sample manifesting a nanowire morphology^37^. **d** SEM image of an electrodeposited WO_3_ film on ITO glass^33^. **e** Structure and morphology characterization of m-WO_3-*x*_ nanowires^36^. **f** Digital photo of an electrochromic device assembled from an m-WO_3-*x*_ nanowire working electrode^36^. **g** Scheme of the rechargeable hybrid Zn^2+^/Al^3+^ electrochromic battery^17^. **h** Multicolor electrochromic display based on a SVO electrode^38^. **i** Electrochromic device assembled by an m-WO_3-*X*_ nanowire working electrode and an ITO glass counter electrode^36^. **j** Hybrid dynamic windows using reversible metal electrodeposition^42^. **k** Reaction schematic showing the electrodeposition of Bi in the absence and presence of Cu. Green and blue circles represent Bi^3+^ and Cu^+^ ions participating here^42^

These inorganic materials have many advantages, such as good chemical stability, fatigue resistance, and an acceptable optical memory (OM) effect, while still facing the following urgent challenges to be solved: high fabrication cost, poor structure modifiability, limited color selection, low pure color quality, slow response rate, and unsatisfactory contrast. All these issues are related to the transport kinetics of both electrons and ions involved in the EC processes, which are affected by the morphology and fabrication of the EC film. To solve these limitations, versatile methods have been developed to prepare materials, including thermal evaporation, laser ablation, sputtering, chemical vapor deposition, spray pyrolysis, electrodeposition, sol-gel techniques, etc.^25–28^. Furthermore, various nanomaterials, such as EC thin films containing nanoparticles, nanorods, and nanowires^29,30^ (Fig. 2a–d), have been realized via simpler and easier methods. These advancements not only reduce the cost but also greatly improve the response speed, cyclic reversibility, memory effect, and other performance parameters^31^. The satisfactory phenomenon benefits from the enlarged contact area between the electrodes and active species, the enhancement of ion migration kinetics, and the electrostatic effect of cations and EC materials. Meanwhile, by optimizing the electrolyte among the conductive layer and the redox active substance in the ion storage layer, the enhancement of the stability of cations in the host lattice of the EC material also helps improve the bistable performance. Mortimer and colleagues^32–35^ have performed excellent pioneering works in this field. Recently, oxygen-deficient monoclinic tungsten oxide nanowires (m-WO_3-*x*_ NWs) were used as EC materials (Fig. 2e, f). In the process of their reversible oxidation state change, Al^3+^, with a small ion radius and a multicharge character, was selected to replace traditional corrosive H^+^ and higher cost Li^+^ as the embedding/de-embedding cation with a triple charge-balancing ability. This improvement endows the EC system with impressive cyclic reversibility (2000 cycles) and a good bistability property (no obvious attenuation within 1 h)^36,37^. Here, diffusion of Al^3+^ into the m-WO_3-*x*_ lattice benefits from the smaller radius of Al^3+^, and the stronger electrostatic effect between the ions and host lattice contributes to the stable existence of the ions in the host lattice.

It is worth mentioning that improving the energy efficiency during the color switching process is also quite meaningful for energy savings, because the current ECDs still require external voltages to trigger color switching. Another effective strategy to save energy is to exploit new ECD structures, such as EC batteries, by utilizing hybrid Zn^2+^/Al^3+^-based electrolytes and WO_3_ electrodes that can retrieve power during the EC process^17,18^ (Fig. 2g). Moreover, many beneficial attempts have also been made to solve the shortcomings of the limited color variation, such as via the color overlay effect of two segments of a sodium vanadium oxide electrode or incorporation of a plasmochromic metal–insulator–nanohole cavity, a photonic Fabry–Perot nanocavity, or others to achieve multicolor displays^13,38^ (Fig. 2h). Although significant progress has been made in inorganic EC materials thus far, the aforementioned technical bottlenecks are still insurmountable and become the key factor restricting their true entry into electronic displays (Fig. 2i).

Controlling the transmittance change of a device through reversible metal deposition and ionization dissociation is another strategy to achieve bistable EC windows^39–41^. The working mechanism is as follows: metal ions are dissolved in a solution with a transparent original state and then reduced to metal atoms by electrical stimulation, and a thin film of metal atoms is deposited on the device surface that affects the transmission of visible light, resulting in each atom acting as a chromophore and jointly affecting the transmittance change of the device. A large transmittance change could be induced with a small thickness EC film due to its dense chromophores. Compared with the traditional EC film, which usually needs to be 100–1000 nm thick, the atomic deposition metal film only needs to be 20–30 nm thick, to achieve sufficiently good transmittance changes with excellent chemical stability and resistance to ultraviolet (UV) degradation. McGehee and coworkers demonstrated a different design of bistable ECDs by reversibly reducing Bi and Cu metal ions, and depositing them on modified ITO to obtain relatively good performance (Fig. 2j, k). Precisely, due to the morphology of the deposited Bi atom, they obtained an ideal black display and its coloration state could be maintained for 25 h without a continuous power supply^42,43^. However, the known systems with reversible ion electrodeposition of Ag, Cu-Pt, and Cu-Ag currently have disadvantages in terms of durability and toxicity, especially when Pt ions are involved. In addition, the limited color selection, low pure color quality, and slow switching speed restrict their potential application in electronic displays.

**Fig. 3:**
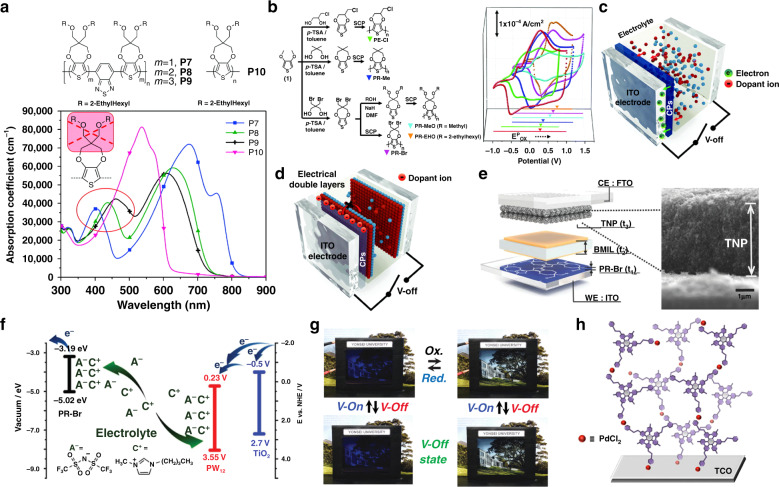
Latest developments in various organic electrochromic materials and devices. **a** Spectral regulation of conjugated polymers based on the “donor–acceptor” approach^48^. **b** Synthetic routes and structure for EC polymers, including PE-Cl, PR-Me, PR-MeO, PR-EHO, and PR-Br, based on the “donor–acceptor” approach, and the corresponding cyclic voltammograms^50^. **c** Schematic diagram of the interfacial electron transfer between ITO and an EC polymer^50^. **d** Schematic diagram of the electrical double layers formed by dopant ions^50^. **e** Schematic diagram of an EC device containing a TiO_2_ layer (BMIL, 1-butyl-3-methylimidazolium bis(trifluoromethylsulfonyl)imide; WE, working electrode; CE, counter electrode; RE, reference electrode)^55^. **f** Mechanism of the charge-balancing effect of the ion storage layer containing TiO_2_ and PW_11_^56^. **g** Photographic images of bistable electrochromic windows^50^. **h** Schematic diagram of the assembly process of organic–metallic hybrid EC materials. TCO, transparent conducting oxide^22^

Organic EC materials, mostly conjugated polymers, have also been reported to enable bistable displays^44–46^. Usually, the applied voltage adjusts the energy level of the subunits of the redox polymer, thereby changing the characteristics of its absorption spectrum and visually showing a color change. The concepts of “band gap engineering” and the “donor–acceptor” approach have proven to be good design principles in using *π*-conjugated polymers for photoelectric devices thus far (Fig. 3a). Reynolds and coworkers^47–49^ have made great pioneering contributions to the field. By modifying the electron-withdrawing/-pushing groups on the polymer backbone, full spectral (color) control can be achieved. Compared with inorganic EC materials, polymer materials have the advantages of light weight, low cost, flexibility, good film-forming ability, and low energy consumption for preparation. Hence, these polymer materials hold great potential for future flexible and wearable displays. Recently, great attention has been paid to improving the device bistability. The known effective strategies are summarized as follows:

- Controlling the spontaneous charge transfer can avoid OM loss in the V-Off state, which is an effective method proposed by Kim and colleagues^50^ to improve the material bistability. Although there are multiple interfaces in an ECD (i.e., Indium Tin Oxide (ITO) electrode/EC film/electrolyte/ITO electrode), fundamentally speaking, the OM loss is mainly related to the charge transfer at the interface of ITO and the EC film. It is greatly affected by the energy levels of the EC polymer. For example, different substituents on the polythiophene skeleton affect its oxidation potential (*E*_ox_) and highest occupied molecular orbital (HOMO) energy levels by changing the dynamic microstructural alterations/supramolecular stabilization/ionization/interactions and electron/charge transfer within and between the polymer chains^51,52^. In general, an electron-withdrawing group increases *E*_ox_ and an electron-pushing group decreases *E*_ox_ (Fig. 3b). Recently, by modifying polythiophene with different substituents (including electron-withdrawing (Br, Cl) and bulky (2-ethylhexyl) side groups), the HOMO level of the polythiophene structure was adjusted below the Fermi level of the ITO electrode (*E*_F_, −4.7 V), thus avoiding spontaneous electron migration between the polymer and the electrode. This is critical to stabilize the polymer state and avoid self-bleaching of its color. When the *E*_HOMO_ of the EC polymer is higher than the E_F_ of the ITO electrode, undesirable interfacial electron transfer (IET) usually occurs reversibly from the EC polymer back to ITO. If the potential difference can be reversed, then the IET process can be avoided, which also enhances the OM (Fig. 3c). However, this method of modifying the substituent on the polythiophene skeleton usually changes its EC color to a certain extent, which affects the color purity. In addition, it is difficult to achieve all the required color changes for electronic displays.
- The OM effect of polymers can be extended by copolymerization of a specific molecular structure^53,54^. Electrochromism of known selenophene polymers occurs in the near-infrared region outside the visible region with good OM characteristics in the colored state. The undesirable color range and good OM are due to the large atomic radius of the selenium atom (Se), which increases the structural hindrance and supramolecular interactions, thus improving the structural stability of related polymer aggregates in the EC state to resist thermal disturbances. On the other hand, thiophene-based polymers with smaller atomic radius sulfur atoms show a large change in transmittance in the visible region and have poor OM in the colored states. Their desirable color range and poor OM are due to the less stable stereostructure and thermal-disturbance-induced instability of their EC states. Therefore, the combination of these two kinds of polymers with different heteroatoms is expected to achieve complementary advantages to improve the bistability of EC polymers. A series of poly(selenophene-co-3-methylthiophene) films were fabricated by electrochemical copolymerization of 3-methylthiophene and selenophene under different feed ratios. Furthermore, their OM in colored states was highly dependent on the monomer feed ratios of selenophene/3-methylthiophene. The known common rule is that the greater the content of selenophene is, the more the optical storage performance is improved. In contrast, the color will be better in the visible region with an increase in the 3-methylthiophene amount. The best result is usually achieved by compromising to find a balanced ratio to achieve a more satisfactory overall performance. Such property optimization usually takes considerable time to obtain a better overall result. Nevertheless, the comprehensive performance is still not satisfactory for electronic displays. At the same time, the toxicity of organic Se has always been a controversial issue, which has hindered its use and development.
- By adjusting the size/amount/structure/properties of the electrolyte and electroactive material in the ion storage layer, the device bistability can also be enhanced within a certain range^55–57^. The classic solid (or semisolid) ECD structure is as follows: ITO electrode/EC layer/conducting layer/ion storage layer/ITO electrode. The electrolyte involved in all three interlayers between the electrodes enhances the electron/charge transfer (conductivity). In addition, the electroactive materials in the ion storage layer have a vital role in balancing the charge of the EC system. Owing to the lower HOMO level of PR-Br (Fig. 3b), the PR-Br-based ECD is capable of maintaining comparatively good bistable states with an electrolyte of ionic liquids (initial unbleached state for several months/bleached state for 1.8 h). However, due to the lack of electroactive material in the ion storage layer near the blank ITO electrode, it is not possible to effectively generate enough counter ions to balance the charges in the system, so the device operating voltage (2.8 V) is still relatively high^50^. Moreover, in the V-Off state, the electrochemical double layer (EDL) near both sides of the electrode spontaneously produces inevitable thermodynamic ion diffusion to reduce the internal electric field and the EC colored/uncolored state cannot be maintained for a long enough time (Fig. 3d). The stability of the EDL is highly dependent on the size and stereochemical structure of the electrolyte ions. By selecting the appropriate ionic liquid electrolyte, a stable ion pair can be formed between the electroactive ions and counter ions to avoid the imbalance of ions on both sides and the related electrostatic repulsion. Meanwhile, by utilizing a TiO_2_ nanoparticle (TNP) layer as the ion storage layer, the potential of the working electrode increases when a certain voltage is applied to the device electrodes^55,58^ (Fig. 3e, f). As a consequence, the ECD including the TNP layer shows satisfactory performance with low overvoltage, long-term bistability, and electrochemical cyclability (Fig. 3g). The above method improves the bistability of the ECD to a certain degree, because the TNPs can effectively stabilize the counter ions they carry, thus avoiding the reverse EC redox reaction caused by charge combination. However, the performance, including the color variation, color conversion speed, and bistability, is still far from satisfactory for electronic displays. There is an urgent need to deeply understand the problems and how to achieve the ideal performance.

Organic–metallic hybrid polymers are formed by complexing metal ions with organic ligands or polymers with coordination sites. Hybrid polymers composed of bis(terpyridine)s and metal ions, such as Fe(II) or Ru(II), show specific colors based on metal-to-ligand charge transfer absorption^59^. Benefiting from metal-centered stability and reversible redox chemistry, organometallic hybrid polymers show superior long-term stability compared to other conjugated EC polymers^60^. Van der Boom and coworkers^60–63^ have greatly accelerated the development of the field. Historically, by changing the metal types or ligands modified with different substituents, versatile color-to-colorless and color-to-color transitions have been demonstrated. To date, significant efforts have been devoted to the effective production of related high-quality coatings and to improving their EC performance (such as coloring efficiency). It was also hoped that a uniform network with high chromophore density could be formed by adjusting the growth process of the relevant EC components (Fig. 3h). Obviously, how to optimize the bistability and the future development of more related materials is worth looking forward to.

Despite the advantages of best-in-class power efficiency and user-friendly color mode, the market’s adoption of reflective electronic display technologies has been very slow. This is because the color obtained by these systems is still poor, the color conversion speed is not fast enough, the color purity is limited and the chemical stability of current materials and devices is not ideal; the “bistable” performance is especially not satisfactory. These intractable deficiencies have severely restricted their further development and application for cutting-edge digital displays. In fact, these issues are highly related to the traditional EC mechanism (a direct-redox-color-changing system), intrinsic structures and fully activated local thermal and electron movement disturbances in the local molecular structure. Significant breakthroughs in structural modification or new syntheses for ideal needs have been complicated and very difficult to achieve thus far. Thus, there is an urgent need to develop a new method to perfect EC properties and extend their application to ideal displays via a simple chemical approach.

## New EC technology overcoming the drawbacks of traditional electronic displays

**Fig. 4:**
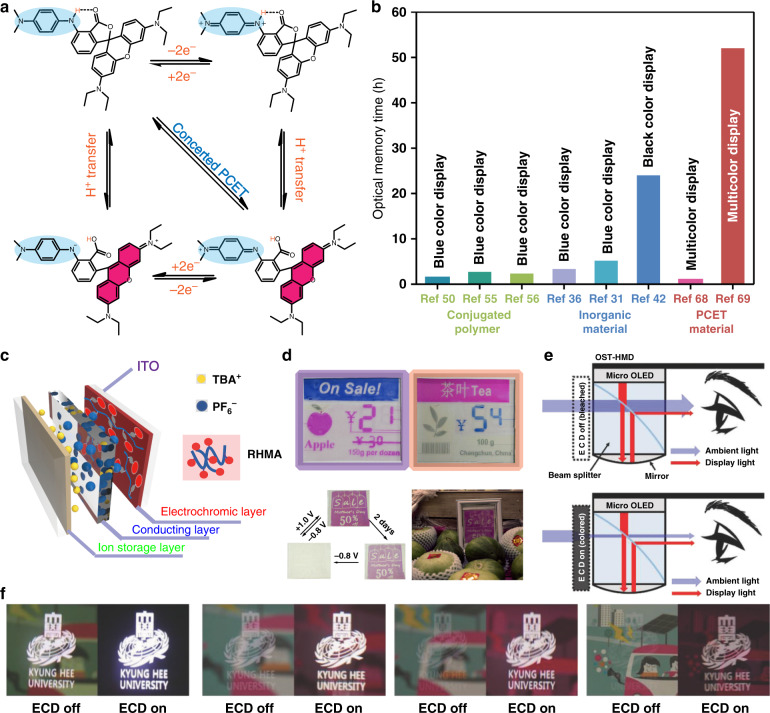
Unconventional design strategies to promote the development of the bistable electrochromic material/device field. **a** EC mechanism based on intramolecular proton-coupled electron transfer^69^. **b** Bistable performance of different types of EC technology. **c** Structure of a bistable EC device. TBA^+^, tetrabutyl ammonium ion; RHMA, EC polymer containing the benzoate-lactone-containing fluoran subunit and the *p*-phenylenediamine moiety^69^. **d** Promising applications of bistable EC devices in energy-saving displays—multicolor electronic label shelf and billboard^68,69^. **e** Mechanism of an ECD regulating the ambient light contrast ratio in an AR system^71^. (**f**) Color gamut and display images of the AR system with the ECD off and on according to different ambient lighting conditions in an office (400 lx), in shade (5000 lx), on a cloudy day (10000 lx), and on a sunny day (15 000 lx)^71^

From the above pioneering works, designing a photonic system possessing ideal color changes and a stabilized molecular state is the key to ideal power-saving bistable displays. To meet the above challenges, Zhang et al.^64–67^ proposed an indirect EC system with a new mechanism—“regulatable electro-acid/base”-induced reversible molecular color switches, overcoming the insurmountable technical bottleneck of traditional direct EC systems. Notably, in their indirect EC system, the coloration units are independent of redox active units, while these subunits specifically interact with hydrogen-bonding groups, mimicking the enzyme reaction centers. In addition, these systems utilize the mechanism of intra/intermolecular PCET in the natural photosynthesis process^68,69^ (Fig. 4a), which avoids unstable free radicals during the reaction via collaborative interactions/stabilization and has been widely used to overcome undesirable energy barriers for various chemical reactions and design functional molecules. Through delicate design of biomimetic molecular switches and supramolecular condensed systems, the redox performance of the molecular “reaction center” and reversibility of color switching were greatly promoted by dynamic self-regulation and charge redistribution of local microstructures. Most importantly, this design avoided the problems of unstable redox states (high entropy energy states) of conventional EC pathways. Thus, it greatly improved the bistability, coloration efficiency, utilization efficiency of external light and electrical energy, and chemical stability of materials and devices (Fig. 4b). Furthermore, through a simplified device assembly process, rapid and reversible color changes in the dynamic state and stable performance in the static state of biomimetic molecular systems in ECDs under electric field induction were realized (Fig. 4c, d).

To broaden the use of this new indirect EC system, Kwon and coworkers^70^ demonstrated a full-color reflective display consisting of different color production units selected as an indirect EC system. Here, to show red, blue, green, and black colors, different leuco dyes were utilized. 2,3-Dimethylhydroquinone (DMHQ), as the redox active species and proton donor compound, could reversibly induce the bleached or colored states of the leuco dye, which was determined by the reaction of the leuco dye with acid species. The novel ECD fabricated with the indirect EC system can easily provide various colors with high brightness and saturation with a broad grayscale. To achieve high transparency in the bleached state and large absorbance in the leuco dye color state over the entire visible region, fluoran derivatives (6’-(diethylamino)-2’-[[3-(tri-fluoromethyl)phenyl]amino]-spiro[isobenzofuran-1(3H),9’-[9H]xanthene]-3-one) were chosen as molecular color switches, because their ring-open state has very good light absorption capacity in the visible region. DMHQ was utilized as the electro-acid here. Hydroquinone accommodates the lactone ring-opening/closing reaction of the fluoran dye through proton transfer under electric stimulation. A demo device of an EC optical shutter (ECOS) was designed and demonstrated by utilizing a fluoran dye, as well as a hydroquinone derivative. It shows outstanding performance with a very high transmittance in its bleached state (82.8% at 550 nm), a low transmittance in its colored state (0.1% at 550 nm), a response time of 1.4 s, and excellent operational stability^71^. An augmented reality display system comprising the ECOS was also fabricated by the authors, which exhibits significantly improved visibility of the displayed image even under strong ambient light conditions due to the excellent visible-light-absorbing function of the ECOS (Fig. 4e, f).

A very promising device of an indirect EC polymer system was recently demonstrated. It is designed by first combining the benzoate-lactone-containing fluoran subunit and the *p*-phenylenediamine moiety to make the desired molecule and then grafting them onto methylmethacrylate polymer chains by an oxyethyl linkage. This new system improves the film-forming properties of its polymer composites by imitating the interwoven structure of peptides^69^. Compared with conventional direct EC materials, the comprehensive performance of this system based on a concerted intramolecular PCET mechanism demonstrates overwhelming superiority. The key performance of its related materials is close to (or even exceeds) the optimum performance of existing EC materials. Impressively, a clear and stable display for more than two days without any refreshing or power consumption is realized. More importantly, this display is similar to a paper book and suitable for reading in bright sunlight. The simplified production/assembly process will also contribute to future industrialization and marketization (Fig. 4d). The above exciting advancements in EC materials and technology will surely inspire and greatly boost the further development of green technology in electronic displays.

## Challenges and future directions for green display technologies

Although the new green technologies of bistable displays have demonstrated super-power-saving performance, they are mostly competing for low-end markets at present. As the market share is not large enough, these green technologies have not yet received widespread attention. The reason is that parts of their performance (such as the speed of turning pages and the color quality/brightness) are not yet comparable to those of mainstream light-emitting electronic display products. Thus, there are two approaches to enable the long-awaited green electronic displays that still meet human needs while using energy efficiently to quickly become widespread in the mainstream market.

One possible solution is to optimize the comprehensive performance of the bistable light-absorbing electronic display to enable it to meet the actual requirements of high-end displays as soon as possible, i.e., to make its key properties, such as the display switching speed, color contrast, cycling times, and chemical stability, fully comparable to those of the existing electronic displays. However, as mentioned above, there are many very difficult technical obstacles in this path.

First, in terms of molecular design, overcoming the oxidative degradation and photobleaching problems of known acid–base dyes is always a challenge^72^. As photobleaching/oxidative degradation is highly correlated with UV and oxygen (O_2_) exposure, it can possibly be solved by exploring better sealing and packaging materials to avoid O_2_ and UV damage. A disadvantage here is that the initial investment in the production plant and equipment will be very expensive, and the oxygen barrier performance requirements of the sealing and packaging materials are also very demanding. Achieving the same goal with low cost and high efficiency is a major challenge in this field. Obviously, we need to go beyond the traditional thinking and technical frameworks. Regarding the protection of dye molecules from UV light, in addition to using traditional packaging materials to absorb and block light, we can explore whether UV light can be effectively converted into visible light by using small molecules or oligomers in the EC layer or packaging materials, i.e., whether the color brightness of EC displays can be enhanced via energy downconversion. Furthermore, we can explore increasing the visible light intensity by energy upconversion to fully utilize the waste energy from heat dissipation of sunlight or in the environment.

The second problem is the limited options for color-changing molecules with different colors and good chemical stability, which is highly related to traditional direct EC systems. For years, researchers have been devoted to the design and synthesis of complex new materials with direct oxidation–reduction discoloration by modifying related substituents and structures to improve the properties and overcome the above shortcomings, but the progress is not satisfactory^73^. Unlike the traditional chemical approach, the aforementioned insurmountable problems could be greatly overcome by indirect EC systems. This new strategy can effectively use hundreds of known molecules as indirect EC colorants, which are easily available, and can be highly economical by selecting desirable colorants with high molar absorption coefficients. In addition to achieving ideal color changes by the “reversible electro-acid/base” method, we can also explore other methods of multicomponent collaborative interaction. For example, additional molecules/oligomers/nanoparticles/quantum dots with color/emission up/downconversion or sharpening characteristics can be used to improve the color purity, quality and brightness of traditional EC/OLED materials. Furthermore, the use of traditional direct EC/electroluminescent and emerging indirect EC or electrofluorochromic systems in the same ECD is also worth exploring. It is possible to obtain complementary advantages and avoid the shortcomings in this way. In addition, the addition/intercalation of free radical quenchers or stabilizers and the avoidance of oxygen ingress can improve the chemical stability of traditional EC materials. Although inorganic materials exhibit outstanding chemical stability and significant progress has been achieved recently, the comprehensive performance still needs to be further improved, to expand their practical applications. First, strategies and materials for achieving more universal panchromatic tunability remain to be further developed, especially for the change from colorless to colorful (“on–off” display mode). Second, although practical methods, such as electrodeposition and sol-gel techniques, have been exploited/developed, there is still a need for further research on large-area and low-cost uniform film preparation technology. Third, it is necessary to further improve the electron/ion transfer rate by introducing nanomaterials into the electrode and EC layer to overcome the bottleneck that the response rate of current ECDs cannot display information satisfactorily.

The third problem is that the EC reaction mechanism and kinetics are still not clear enough. The influence of the system microenvironment on the EC reaction, such as the types of solvents, electrolytes, and additives, temperature and pH, needs to be further characterized and observed via various in situ tests. The synergism in the PCET reaction, the dynamic processes of the electrons and ions in the inorganic/organic EC reaction, the electron transfer between the electrodes and materials, and other processes involved in the EC reaction, all need to be understood more deeply and optimized accurately and intelligently.

The fourth problem is highly related to device assembly. Compared with LCDs/OLEDs, the color of current EC displays is not bright enough and their page-turning speed is slow. The main reason is that the contact area between the flat electrode and the EC molecules is limited. Current EC systems utilize electrolytes as charge carriers for redox of color-changing molecules. The diffusion rate of known electrolytes is not fast enough, especially in solid EC thin films. The color brightness of EC displays is highly related to the amount of color-changing molecules (or subunits of redox polymers), which is highly related to the thickness of the EC thin film. In contrast, the page-turning speed of EC displays is inversely related to the device thickness^69^. Even though new electrolytes for better charge transfer have been a long-awaited dream, success has been very limited thus far. As the size of electrolytes cannot be small enough, the weak intermolecular interaction between the known electrolytes and media greatly slows the diffusion rate and charge transfer speed. Thus, significantly increasing the contact area between the electrode and EC molecules, as well as the electron and charge transfer efficiency in solid or semisolid films, is an extremely important future research direction. The traditional planar ITO structure needs to be replaced by a new type of electrode that has a highly conductive three-dimensional (3D) structure and excellent transparency. How to long-term stably and tightly connect EC materials to the electrode surface through chemical or physical methods is also a subject worth exploring. At the same time, the ability to form a protective layer with switchable ion/electron channels on the surface of the EC material can not only ensure the smooth transmission of ions or electrons during the EC process but also effectively prevent self-fading or self-discharge when there is no electric field.

The primary goals are further improvement of device fabrication technologies, and further development and utilization of needed auxiliary materials. In addition, the compatibility of the above new materials and technologies with mainstream display technology is the key to their rapid market entry, and the integration of old and new display technologies is a future direction. In particular, we need to explore whether we can use known/new electron and hole carriers in OLEDs to speed up the EC speed. We should explore speeding up of the diffusion rate of electrolytes via media modification to increase the micro/nanoporosity of EC thin films via known technologies of new structural design and molecular 3D self-assembly, metal-organic frameworks, freeze-drying, hydrogels, etc. We should also explore significantly increasing the contact area between the electrode and EC molecules via realization of nanorange 3D electrode networks via laser etching, 3D printing, self-assembly, thermal healing, electrospinning, etc. It is worth mentioning that the advantages of nanomaterials, such as nanowires, nanorods, nanobelts, nanofibers, and nanoparticles, include a large electrolyte and electrode contact area, high electrical conductivity, a short ion diffusion route in the diameter direction, and efficient electron transport in the length direction. As the ion/electron double injection distance can be significantly reduced in this way, the switch response speed will be improved to achieve satisfactory results. Nevertheless, the size, dimensionality, shape, and structure of these nanomaterials should be delicately designed and controlled. On the one hand, the specific surface area and porosity of the materials should be improved by fabricating nanostructures. On the other hand, due to the high density and small pore size of some nanomaterials, the important criterion for selecting and using them is to determine whether they hinder ion migration. As nerve systems in nature likely use the stimulus response collaborative actions of dynamic alteration of their 3D structures and ionization to transfer electrons and charges, their use would be a very mild and instant strategy that effectively avoids steric hindrance and heat loss during electrolyte diffusion in known EC, battery, capacitor, and other electrical systems. Thus, we should learn in depth from nature, explore the possibility of man-made “nerve networks” systems^74^ via structural design, synthesis, and molecular self-assembly, and attempt to use bionic “nerve networks” to replace the current method of electrolyte diffusion for quick transfer of electrons and charges in the system. The success of this will likely revolutionize modem mainstream electronic displays, energy storage, and other technologies, and overcome the long-puzzled technical bottleneck of electrolyte diffusion. Meanwhile, the development and combination of more advanced in situ spectroscopy techniques, such as in situ electron microscopy and in situ nuclear magnetic resonance, to deepen the understanding of state changes of structures during the reaction process will also contribute to the precise design of bistable EC materials.

The other possible solution is to revolutionize the mainstream light-emitting display technologies and make them partially (or fully) bistable and super-power saving. To realize this seemingly “impossible” dream, revolutionary technologies combined with new mechanism design and device structure optimization are clearly needed. We should explore whether we can delay their light emission or extend the lighting time with limited electrical stimulation via new materials and unusual device structure designs. For example, we can use existing known mechanisms, such as Förster resonance energy transfer^75^, photoelectron transfer, aggregation-induced luminescence^76^, phosphorescence fluorescence reversal^77^, and PCET, to effectively delay and extend the light emission time. Important progress has also been made in related fields, such as long-persistent luminescence (LPL) materials with the ability to store and slowly release excited-state energy^78^. In addition to improving the efficiency of exciting triplet states, it is equally important to protect triplet states to prolong their luminescence lifetime. Through the use of inorganic/organic crystals, quantum dots, metal-organic frameworks, H-aggregates, functionalized/hybridized/defective small/large condensed ring conjugated molecular systems, and others, significant progress has been made in related research in this area to date. Recently, an organic LPL long-acting luminescent material was fabricated by using triphenyl quaternary ammonium phosphorus TPP-3C2B as an organic electron trap. The cationic triphenylphosphonium core acts as a strong electron acceptor for the photoinduced charge transfer event and can also serve as a protective trap to stabilize and protect the excited free radicals so that the system can slowly reorganize with long-lasting luminescence characteristics, with a luminescence duration of up to 7 h. In addition, we can also adopt strategies involving cross-fusion with existing technologies, such as electrofluorescence, electrophosphorescence, controllable delayed (or long-lived) electroluminescence, and indirect EC molecular switching. We can attempt to explore whether to convert current direct LEDs/OLEDs into indirect LEDs/OLEDs, EC-LEDs/OLEDs, etc. Apart from saving energy by exploring the device bistability, increasing their luminous efficiency is an alternative method to save energy. To date, the luminous efficiency of LEDs/OLEDs is very low, mainly due to their internal resistance and heat dissipation. Thus, altering the display modes of LEDs/OLEDs or coupling metal/nonmetal (i.e., graphene, carbon nanotube, carbon 60/70/80, conducting polymer, etc.) conductors or bionic electronic 3D “nerve networks” to the systems might be good alternatives to effectively reduce the internal resistance and heat dissipation and increase the luminous efficiency. Moreover, the role of supramolecular weak bonds, such as hydrogen bonds, in material design and structural stability cannot be ignored. The integration of various known or new display techniques will likely offer a promising strategy to satisfy various human needs, such as EC-LED/OLED dual mode systems for all-weather ultra-power-saving display technology.

In addition to traditional EC windows, bistable display technology involving electrochromism is gradually being applied in energy-saving electronic price tags, billboards, rearview mirrors, and artificial irises to adjust the camera aperture. By preparing reflective ECDs, we can exploit electronic paper products with multicolor displays and repeatable erasure, which can greatly save energy and avoid waste and consumption of paper. Apart from serving people’s daily life needs, novel electronic materials have wide cutting-edge applications, such as in the field of molecular computers with information storage capability. Combined with flexible substrates, such as fibers and polymers, the bistable electronic materials with full-color adjustable properties are promising in active and controllable military camouflage. ECDs with transparent display performance have great potential in the fields of augmented visual reality, head-up display and artificial intelligence. Overall, bistable electronic display products are not only crucial for the future of intelligent life but also can drive the related high-tech industry.

**Fig. 5:**
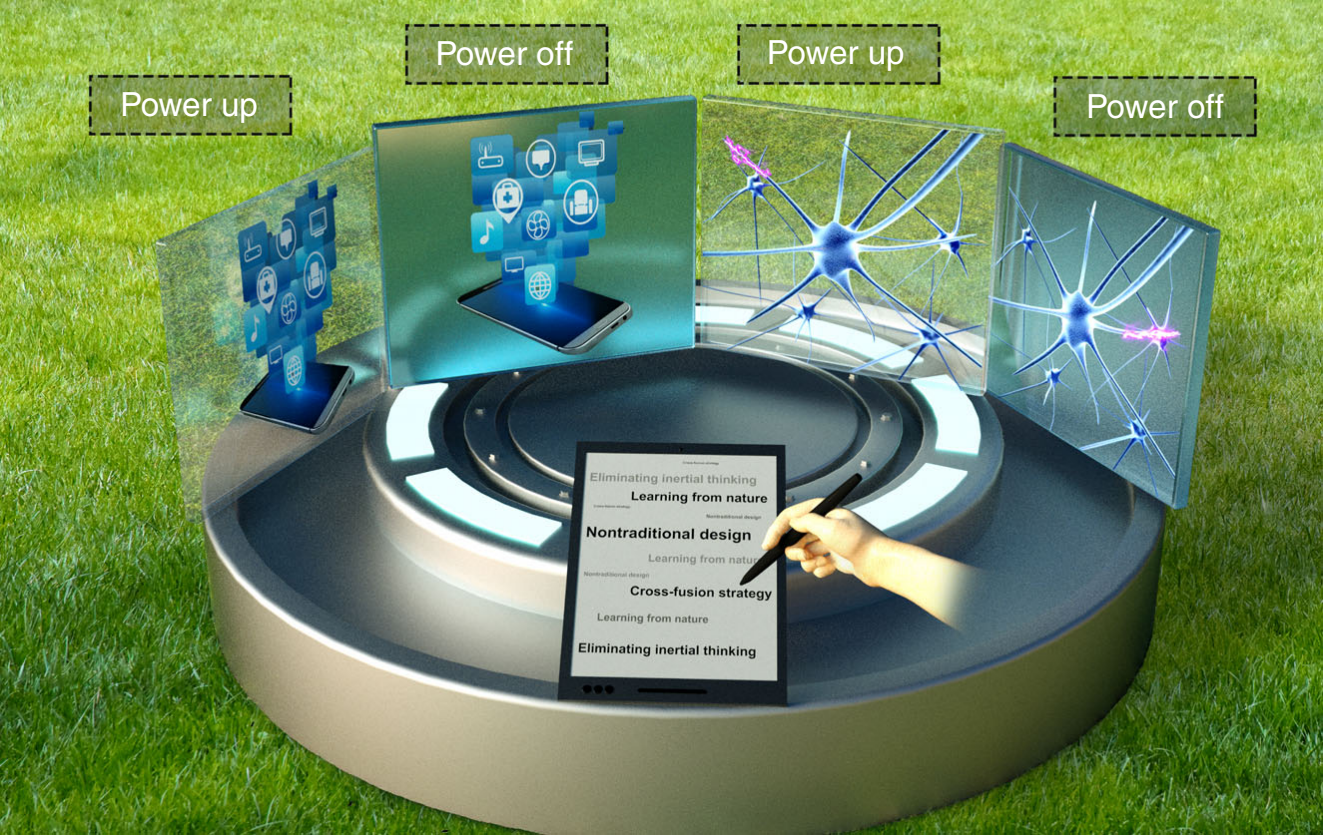
Research strategy and development of green technologies in the future. Scheme of the green display technologies combined with untraditional strategies

Despite the heavy responsibilities, we foresee a bright future. We are convinced that future super-power-saving bistable displays will not only belong to the field of known EC and electrophoresis technologies but also appear in the field of electroluminescence and others. History shows that the driving force behind scientific and social progress is the spirit of doubt and challenge. Many seemingly difficult problems are gradually resolved by further understanding the cause of the problem through the collaboration of science and technology workers. Countless facts exemplify that many dreams in the world are no longer impossible, but people do not dare think about or are not willing to put effort into achieving them. Essentially, traditional thinking and restriction of cognitive abilities hinder people’s inventiveness and creativity. Once the restraint of thinking that certain achievements are “impossible” is broken, various miracles will continue to be realized by humans via magically “possible” explorations. Hopefully, in the near future, we can overcome the above bottlenecks through cooperation with an increasing number of visionary researchers and industry to optimize the green technologies of bistable displays and promote their global commercialization (Fig. 5).

## Opportunities and prospects for a green technology revolution

Finally, we would like to note that the aforementioned problems regarding electronic displays and possible solutions epitomize the rapid development of modern society. The problems of very high power consumption and low energy utilization efficiency widely occur in all human activities, such as those involving air conditioners, refrigerators, electric cars, computer networks, and automation control systems. For instance, the current power consumption of global lighting is 20% of global electricity generation. Meanwhile, the electro-optical conversion efficiency of lamps is extremely low, only 5–20% of the theoretical conversion rate, with the vast majority wasted via heat dissipation. We must perform in-depth studies to find the root cause of the high resource consumption and low energy utilization efficiency and improve this situation by changing the existing mode of technology development. In this spirit, a few examples are presented as follows: (i) we could mimic the light production of fireflies to achieve highly efficient chemiluminescence. The energy conversion efficiency of lighting is currently extremely low in the multiprocess of chemical energy→heat→mechanical force→electricity→light. We could increase the energy conversion efficiency significantly if we can find a better method to generate light from chemical energy (or heat) directly and efficiently. The known chemiluminescence seems to involve a functionalized aromatic structure, which likely acts as a relatively rigid energy transfer center to assist electron transfer and light emission and reduce heat loss. We should learn from nature and explore the possibility of increasing the light-emitting efficiency by introducing partially functionalized aromatic derivatives into the lighting systems of LEDs, OLEDs, thermo-lighting, chemi-lighting, etc. Hopefully, similar to the firefly, we could generate light at low temperatures and create a “miniature sun” via the effective use of geothermal radiation, the heat lost from fossil fuel power plants, etc. In addition, we could create an effective low-temperature thermo-lightning system to utilize heat radiation in nature via two-photon/multiphoton integration and blueshifted light conversion with the help of bionic aromatic platform carriers. (ii) We could use the synergy of the resonance field effect and/or quantum resonance wave effect of high-quality LEDs/OLEDs, optical fibers, laser systems, or others to achieve light delivery without (or with low) energy loss. (iii) We could use super-power-saving EC materials as indoor/outdoor decorations and smart windows that are capable of energy storage and release or power generators/battery chargers.

As long as we are actively learning from nature, advancing the understanding of its working principles in depth, applying what we newly learned, and questioning and challenging traditional knowledge, we will become “masters of creating miracles” and make many seemingly “impossible” achievements “possible”. With the new generation of EC or EC-LED/OLED materials and technologies, in the near future, the glamour of electronic display use will no longer entail environmental issues. Hopefully, this study can allow an increasing number of researchers to understand the above scientific and technological challenges and their significance, trigger global cooperation to challenge the “impossible” scientifically forbidden zone, and bring innovation in green technology to a new level.
